# International Medical Congress

**Published:** 1881-09

**Authors:** 


### INTERNATIONAL MEDICAL CONGRESS.

*The proceedings of Section XII. (Diseases of the Teeth), opened at 3 p. m., on Wednesday, the 3rd inst., with the following Address from the President, Mr. E. Saunders.*

Gentlemen—Before assuming the duties and responsibilities of President of the Odontological Section of this great Congress, permit me to thank you for the honor you have conferred upon me in electing me to that high office; and let me assure you that it will be my earnest endeavor to justify your choice, however I may fail to satisfy my own, and your well-warranted expectations. And, indeed, were it not that I am sustained by the kind co-operation of two Vice-Presidents and a Secretary, in whom our profession feels a just and legitimate pride, I might well feel oppressed by the onus of doing full justice to the position on so august an occasion. Nor was it, I am free to admit, until I had the permission of the Executive Committee to call around me, as a Council, some old and tried associates in professional work, that I began to experience the requisite energy and confidence. And having made this personal avowal and confession, I turn to the distinguished company before me, who have done us the high honor to visit our too great but not too gay capital, and to become for a short time, and for our signal profit, our guests at this great intellectual banquet.

Et a vous, Messieurs les Etrangers, je desire, au nom des Membres Anglais du Congress, au nom de notre plus grande Societe La Societe Odontologique de la Grande Bretagne, au nom de la profession meme en Angletere, vous souhaiter la bien-venue. Croyez, Messieurs, que nous sommes tres sensibles de l'honneur que vous nous avez fait en visitant a ce moment notre trop grande, mais pas trop gaie ville de Londres, et cryez que c'est notre desir de vous rendre la visite, a une fois, agreable et fructueuse.

Permit me, gentlemen, on behalf of the English Members of the Congress, on behalf of our largest Society, the Odontological Society of Great Britain, now in the twenty-fifth year of its somewhat chequered, but on the whole, prosperous existence, and on behalf of the whole body of the Dental Profession in England, to offer you a hearty welcome, and to assure you of our earnest desire to render your too brief visit both profitable and pleasant. We hail the happy occasion of our friendly interconrse, and we trust that friendships begun under such benign auspices may continue and progress in interest through many prosperous years. Such gatherings as these, with the pleasureable amenities they involve, do much to soften and refine the manners, to quicken the intellect of all concerned, and to remove misconceptions of national or individual character, which are apt to be engendered by isolation and want of friendly intercourse. Congresses, such as that at which it is our privilege at this time to assist, serve a great social purpose apart from the intellectual and scientific aims at which they are more immediately directed. It is much that they afford an opportunity and a stimulus for intellectual effort, which might otherwise, with man's proverbial procrastination, never be called into action. But in these days of an ever-teeming press, and of facilities for the free intercommunication of ideas, this is subordinate to the advantage of personal knowledge of the individual, and the living interchange of thought. The modern Congress, which seems now in high favor, owes its existence, or shall it not be said its revival to the intellectual activity, joined with a wide eclecticism, which is a characteristic of our times, and which seeks to assimilate to itself whatever is of value in the past, or in other lands, whether in social manners and customs, in matters of dress and daily life, in schools of architecture, or in the realms of science or art. The general accepted idea of a Congress is, if I mistake not, more than a fortuitous assembly of persons engaged in similar pursuits and drawn together by community of of thought and interest. It is the deliberate coming together of distinguished men or of experts, of set purpose and for a specific object; the persons constituting the Congress being invited and selected with a due regard to their knowledge of the subject, for the consideration of which it has been convoked. This is especially evident in political affairs, and notably in a recent instance in a northern capital, where the great countries of Europe, represented by their most distinguished statesmen and diplomatists, met in solemn conclave for the rectification of national frontier lines, and for the determination of other questions of vital importance which must otherwise have been left to the rude arbitrament of the sword. And may we not indulge the hope, in the interests of an enlightened humanity, that this high function may be more extensively employed to interfere between the unbridled ambition and lawless rapacity of States, more powerful than just, and the resolute resistance to subjugation or spoliation on the part of the oppressed, protracted, it may be, to the bitter end through years of carnage and misery? So might the world be spared the saddening spectacle of "man's inhumanity to man," and the fair page of contemporary history be unsullied by the foul blot of the blood-stained record. Ours, however, is a Congress of peace, and we are happily not called upon to compose animosities or to adjudicate upon conflicting claims. The triumphs which we are met to celebrate are those of man's skill in limiting and repairing the ravages of disease; our victories, those over nature herself, when she is forced to yield up another of her secrets as the reward of patient research or of well-conducted experiment. Our international reunion may be regarded as a periodical taking stock of the gains of science, of improved appliances, of more accurate means of diagnosis, and of more efficient modes of treatment in the various departments of medical practice. And for the better and fuller carrying out of this intention, it has been found advisable, having regard to the present advanced state of medical and surgical science, to divide the work of the Congress into sections.

These sections carrying on their work simultaneously, and the work of each section being complete in itself, greater fulness and accuracy of detail are secured without any sacrifice of the unity of the one grand result, as the work of each section is the necessary complement of the whole. In that with which we are more immediately concerned, one of the youngest departments of surgical practice, and which for the first time enjoys its own distinct and prominent position, considerable interest will attach to the question of education and the regulations which in each country govern the entrance into the profession. The former part of this very important subject will, I trust, shortly be brought under the notice of the Congress by one than whom none is at once more fully informed, and more entitled to speak with authority, I mean Mr. John Tomes, who has in this direction during the last quarter of a century done much to advance the interests, and to establish a strong claim on the gratitude of the profession. Nor would the English sense of justice and fair play be satisfied without an acknowledgement of the more recent services of his colleague in this good and great work, Mr. James Smith Turner, without whose unsparing devotion of time and energy it could not have been brought to so successful an issue. To the joint action of these two gentlemen; to the lawyer-like precision and forethought of the former no less than to the vigilance and promptitude of the latter, is the profession indebted for that invaluable piece of legislation, the Dentists' Act of 1878. It must not be forgotten, however, that to our foreign friends, the nature of the enactment is of greater interest than the means or the persons by whom it was obtained. By the provisions of this act, then, which came into operation on August 1, 1879 it is forbidden to any one, to use the word Dentist, Dental Practitioner, or other title implying that he is qualified to practice Dental Surgery, unless his name appears on the Register of that body; thus giving to the Dentist the same protection and privileges as are enjoyed by the Physician and Surgeon in this and other countries. By this measure the opprobium was removed which so long rested on the Dental Profession in this country, that it included a large proportion of ill-qualified practitioners, and in many cases persons who were unsuccessful in other pursuits, and who were attracted to it by the absence of restrictions or of preliminary examination. By the provisions of this Act, introduced by Sir John Lubbock, not only are the public preserved from the extortion and malpraxis of the ignorant and unprincipled, but a grave discouragement is removed from the educated and honest practitioner. For it is only in human nature that high aims and honest zealous work should languish in the atmosphere of indifference and lack of appreciation. In thus obtaining legal sanction for the organization of the profession it was desired strictly to maintain its connexion with the Royal College of Surgeons, as it was rightly felt that separation from that body would involve abdiction of the status which it had hitherto enjoyed. And when the college had been memorialised on the subject, shewing that the curriculum for the diploma for general surgery, which was the only qualification then open to him, did not comprise certain matters of the first importance to the Dental practitioner, that, in fact, the entire subject of Dental Surgery found no place either in the teaching, or at the examining board, an arrangement was accepted for more fully meeting the requirements of the case. Accordingly a conjoint board of examiners, consisting of half surgeons and half specialists, was created for the licentiateship of Dental Surgery, with a corresponding modification of the prescribed course of study, eliminating much that was of little value, and substituting what was regarded as specially necessary in that particular line of practice. Thus, by varying, not by lowering, the educational standard, an arrangement has been effected, which if not in all respects perfectly satisfactory, goes far to meet the reasonable views and wishes of those who have the welfare of the profession at heart. With this bare outline of our proceedings in reference to the organization of the profession before us, we shall listen with interest to what has been accomplished in other countries in the same direction, not, it may be hoped, without mutual profit and advantage. Gentlemen, I feel that I ought no longer to tax your attention, but having declared this section of the Congress open, that we shall prepare ourselves to listen with appreciation and enjoyment to those varied and valuable contributions to the literature of our speciality with which we are so liberally favored both from home and foreign sources. And first your attention will be asked for the always welcome utterances of one whose contributions to science during a long series of years, many of them having a direct interest for our own speciality, and almost unparalleled for number and value, have made his name a household word in both hemispheres. I mean Professor Owen. We feel grateful for his presence here to-day, which will confer prestige on our proceedings, and we tender him our thanks, with our sincere felicitations that he has been able to witness, in unimpaired health and energy, the realization of his hopes and wishes in the completion of that noble structure, the Museum of Natural History.

